# Complete Genome Sequence of Sphingobium barthaii KK22, a High-Molecular-Weight Polycyclic Aromatic Hydrocarbon-Degrading Soil Bacterium

**DOI:** 10.1128/MRA.01250-20

**Published:** 2021-01-07

**Authors:** Jiro F. Mori, Robert A. Kanaly

**Affiliations:** a Graduate School of Nanobiosciences, Yokohama City University, Yokohama, Japan; Loyola University Chicago

## Abstract

Sphingobium barthaii KK22^T^ is a high-molecular-weight polycyclic aromatic hydrocarbon-degrading soil bacterium that has been investigated in biotransformation, microbial ecology, and DNA damage studies. The complete genome sequence of S. barthaii revealed four closed circular sequences, including two chromosomes, a megaplasmid, and a smaller plasmid, by hybrid assembly using short- and long-read sequencing technologies.

## ANNOUNCEMENT

The Gram-negative alphaproteobacterium Sphingobium barthaii KK22^T^ has been investigated (i) for its capabilities to biotransform high-molecular-weight (HMW) polycyclic aromatic hydrocarbons (PAHs), (ii) for its major role in a hydrocarbon-degrading bacterial consortium, and (iii) as a model organism in DNA damage studies (1–7). It was isolated from a diesel fuel-grown bacterial consortium that originated from cattle pasture soil from the Gulf region of Texas (1, 8, 9). Some sphingomonads may possess a second chromosome in addition to multiple plasmids, including large (>100-kbp) plasmids or “megaplasmids,” that are potentially responsible for the catabolic flexibility of this bacterial group (10). The draft genome sequence of S. barthaii was announced in 2013 (11); however, functional gene distributions on the chromosomes and plasmids were not able to be determined. Now, the new information presented here shall not only provide clear insights into the metabolic capabilities of S. barthaii but also support its potential biotechnological applications in bioremediation and green chemistry (12).

Here, a hybrid assembly using short-read (DNBSEQ-G400; MGI Tech, Shenzhen, China) and long-read (GridION X5; Oxford Nanopore Technologies, Oxford, UK) sequencing technologies was employed to close the chromosomal and plasmid sequences of S. barthaii. S. barthaii that had been grown for 4 days on 300 mg liter^−1^ phenanthrene in Stanier’s basal medium at 30°C with rotary shaking at 150 rpm in the dark (7) was subjected to genomic DNA extraction using the DNeasy PowerWater DNA isolation kit (Qiagen, Hilden, Germany). For the DNBseq analysis, genomic DNA was sheared to average 400-bp fragments with an S2 device (Covaris, Woburn, MA, USA), and the DNBseq library was prepared using the MGIEasy universal DNA library preparation kit (MGI Tech) according to the manufacturer’s instructions. A total of 20,576,138 reads with a 150-bp paired-end read length were sequenced with the DNBSEQ-G400 platform and were subjected to trimming and quality filtering using Cutadapt (v. 2.7), SeqKit (v. 0.11.0), and Sickle (v. 1.33). For the GridION analysis, 1,000 ng of genomic DNA was barcoded using native barcoding expansion (Oxford Nanopore Technologies), and the library was prepared using a ligation sequencing kit (SQK-LSK109; Oxford Nanopore Technologies). A total of 457,744 reads with an average length of 6,451.8 bp and an *N*_50_ value of 10,186 bp were generated by the GridION platform using R9.4.1 flow cells and Guppy (v. 4.0.11) for live base calling. The raw reads were subjected to trimming and quality filtering using Porechop (v. 0.2.3) and Filtlong (v. 0.2.0; minimum length of 1,000 bp), and error correction was performed using Canu (v. 2.0). All software was used with default settings unless otherwise specified.

After the trimming and quality filtering of the raw reads, *de novo* hybrid assembly was performed using Unicycler (v. 0.4.7) (13), and the assembly was validated with Bandage (v. 0.8.1). The complete genome sequence thus determined (4,984,172 bp, with 787.5× coverage) consisted of two circular chromosomes (3,389,351 and 1,187,212 bp), a circular megaplasmid (387,592 bp), and a circular small plasmid (20,017 bp) with an overall G+C content of 64.7%. Gene annotation by the NCBI Prokaryotic Genome Annotation Pipeline (PGAP) (v. 4.13) identified 4,623 coding sequences (CDSs), 9 rRNAs, 54 tRNAs, and 1 transfer-messenger RNA. Among the CDSs, 3,220 (69.7%) and 989 (21.4%) sequences were identified on the large and small chromosomes, respectively, while 391 (8.5%) and 23 (0.5%) sequences were identified on the megaplasmid and the small plasmid, respectively.

Notably, it was found that all seven sets of aromatic ring-hydroxylating dioxygenase (ARHD) genes encoded in the S. barthaii genome (2) were located on the megaplasmid, with six sets being tightly clustered. This ARHD gene cluster is shared among some aromatic hydrocarbon-degrading sphingomonads and is considered to be responsible for biotransformation of various xenobiotic aromatic hydrocarbons (14, 15). However, due to the incompleteness of the sphingomonad genomes previously sequenced, there is only one *Sphingobium* genome (S. yanoikuyae SJTF8) available in public databases for which the ARHD gene cluster was reported, also located on its megaplasmid. The genome presented here is the second report of a *Sphingobium* megaplasmid that carries the ARHD gene cluster, and these results support the hypothesis that transfer of these plasmids in sphingomonads is of major importance in regard to biodegradation of aromatic hydrocarbons including HMW PAHs (10).

### Data availability.

The complete chromosome and plasmid sequences of S. barthaii KK22 were deposited in NCBI GenBank under the accession numbers CP060035, CP060036, CP060037, and CP060038 and in the Integrated Microbial Genome and Microbiomes (IMG/MER) database (Joint Genome Institute, IMG accession number 2883162986). The raw sequences are available from SRA accession numbers SRR12950076 and SRR12950077 under BioProject number PRJNA655586 and BioSample number SAMN16591971.
